# Effects of artificial sweeteners on coronary heart disease: A 2-way 2-sample Mendelian randomization study and mediation analysis

**DOI:** 10.1097/MD.0000000000047656

**Published:** 2026-03-13

**Authors:** Shu Sun, Yonggeng Zhang, Song Yi

**Affiliations:** a Cardiology Department, Yichun People’s Hospital, Cardiovascular Medicine, Yichun, China.

**Keywords:** artificial sweeteners, coronary heart disease, insulin sensitivity index, mediation analysis, Mendelian randomization

## Abstract

This study aims to investigate the causal relationship between intake of artificial sweeteners and coronary heart disease (CHD) and identify and quantify the role of insulin sensitivity index as a potential mediator. Using summary-level data from genome-wide association studies, a 2-sample Mendelian randomization (MR) analysis was conducted on genetically predicted artificial sweeteners (64,949 cases) and CHD (1,85,000 cases). In addition, we used a 2-step MR to quantitatively determine the proportion of the effect of genetically predicted intake of artificial sweeteners on CHD mediated by insulin sensitivity index. The MR analysis determined that a higher genetically predicted intake of artificial sweeteners (main MR analysis odds ratio: 1.320 per SD increase, 95% confidence interval: 1.044–1.668) increases the risk of CHD. There is no strong evidence suggesting that genetically predicted CHD has an impact on the risk of intake of artificial sweeteners (odds ratio: 0.996, 95% confidence interval: 0.990–1.002). The proportion of genetically predicted CHD mediated by insulin sensitivity index is 19.61%. In conclusion, our study identifies a causal relationship between intake of artificial sweeteners and CHD and that insulin sensitivity index can serve as its mediator. In clinical practice, excessive intake of artificial sweeteners should be avoided in CHD patients and susceptible individuals.

## 1. Introduction

Artificial sweeteners, commonly used as substitutes for sugar, have been a subject of debate regarding their health effects, particularly on cardiovascular diseases such as coronary heart disease (CHD). CHD, a condition characterized by the narrowing of coronary arteries supplying blood and oxygen to the heart, is a leading cause of mortality worldwide.^[1,2]^ The pathogenesis of CHD involves a complex interplay of genetic, environmental, and lifestyle factors, including dietary influences. One potential physiological mechanism by which artificial sweeteners may affect CHD is through their impact on metabolic processes. Some studies suggest that artificial sweeteners could disrupt the body’s normal glucose and insulin regulation.^[3,4]^ This disruption may lead to insulin resistance, a condition that is associated with an increased risk of CHD. For instance, artificial sweeteners may interfere with the signaling pathways that regulate insulin release and sensitivity, potentially resulting in abnormal blood sugar levels and increased lipid accumulation in the arteries. Another potential mechanism is related to inflammation. Artificial sweeteners may trigger an inflammatory response in the body.^[5,6]^ Chronic inflammation is a critical factor in the development of atherosclerosis, which is the underlying process of CHD. Inflammation can damage arterial walls, thereby promoting the formation of plaques that narrow the coronary arteries. Recent research has indicated a potential link between the consumption of artificial sweeteners and an increased risk of developing CHD. A study published in the British Medical Journal found that participants who consumed artificial sweeteners had a higher risk of cardiovascular events compared to those who did not, adjusting for confounders such as age, sex, and known cardiovascular risk factors.^[7]^ However, the mechanisms underlying this association remain unclear.

Observational studies have produced mixed findings regarding the association between artificial sweetener consumption and the risk of CHD. Some studies suggest a positive correlation, while others report no significant relationship. These investigations frequently rely on self-reported dietary intake or concentrate on specific sources of artificial sweeteners, such as low-calorie beverages, without considering the broader dietary context. This limitation highlights the necessity for more comprehensive assessments of artificial sweetener intake and its potential effects on CHD. The NutriNet-Santé Cohort study provides a robust prospective analysis of the relationship between artificial sweetener intake and hard endpoints of CHD, including CHD.^[8]^ The study also identifies significant gaps in the current research, particularly concerning the relationship between artificial sweetener intake and specific subtypes of CHD, as well as CHD mortality. Furthermore, the potential modifying effect of genetic susceptibility on this relationship remains unexamined, despite established interactions between genetic factors and lifestyle choices in influencing disease risk. The recent statement from the World Health Organization underscores the necessity of accumulating additional evidence prior to the formulation of guidelines regarding the use of non-sugar sweeteners. This call for further investigation is especially pertinent in light of the widespread consumption of artificial sweeteners and their potential implications for public health. Additionally, the relationship between artificial sweetener intake and type 2 diabetes mellitus, a known risk factor for CHD, warrants closer examination.^[9,10]^ Understanding the extent to which type 2 diabetes mellitus mediates the effect of artificial sweetener intake on CHD is essential for elucidating the underlying mechanisms and informing dietary recommendations. Although current evidence indicates a potential association between artificial sweeteners and CHD, additional research is necessary to establish a causal relationship and to further comprehend the mechanisms involved. Mendelian randomization (MR) analysis, which uses genetic variation as an instrumental variable (IV) to evaluate potential causal relationships, could be a valuable approach in this context.^[11]^ Additionally, mediation analysis may elucidate the pathways through which artificial sweeteners influence CHD, particularly by examining insulin-related indicators as mediators. In conclusion, the relationship between artificial sweeteners and CHD remains an area of active research. While preliminary studies indicate a potential association, further investigation is essential to clarify the underlying mechanisms and to establish causality. This research could have substantial implications for public health and dietary guidelines.

## 2. Materials and methods

### 2.1. Study design

**Figure 1.**
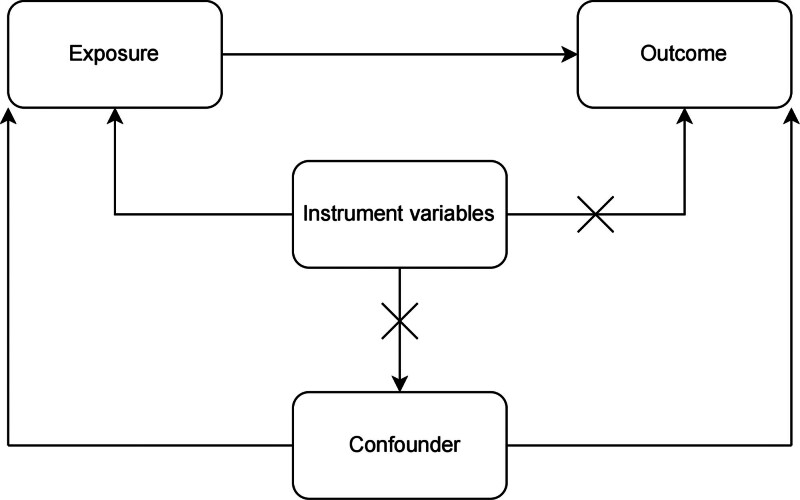
Mendelian randomization analysis flowchart.

The data utilized in our analysis is publicly accessible and has received approval from the institutional review board for the relevant studies. Consequently, no further sanctions are required. All generated results are presented in the article and its supplementary materials. In this research, we examined the causal relationship between artificial sweeteners and CHD using a 2-sample 2-way MR approach (Fig. 1). In our study, single nucleotide polymorphisms (SNPs) are designated as IVs.^[12]^

### 2.2. Data sources

The data used in our study are all publicly available. Moreover, the participants in the Genome-Wide Association Studies (GWAS) are all of European ancestry. The research data related to artificial sweeteners are from the study by Ben Elsworth et al, which includes 64,949 cases. The insulin sensitivity index is from the meta-study by Walford et al, which includes 16,753 cases and 24,12,427 SNPs.^[13]^ Insulin sensitivity was represented by the Modified Stumvoll Insulin Sensitivity Index, as defined by Walford et al, which incorporates fasting insulin, 120-min insulin, and 120-min glucose levels obtained during an oral glucose tolerance test. The data on CHD are from the meta-study by Nikpay et al, which reports a GWAS meta-analysis of 1,85,000 CHD cases and controls.^[14]^ The research design, including sample collection, quality control procedures, and estimation methods, has been detailed in the original publications. Importantly, all GWAS data originate from distinct consortia or organizations, ensuring there is no overlap in the samples utilized.

### 2.3. IVs selection

To estimate causal effects using genetic variations, three basic assumptions of IVs must be satisfied: the IV is associated with the exposure factor; the IV is independent of confounding factors; and the IV is unrelated to the outcome variable and acts on the outcome variable only through the exposure factor.^[15]^ Specifically, the IVs in this study were screened to meet the following conditions. When there are too few genome-wide significant loci in the original GWAS results, a genome-wide significance threshold of *P* < 5 × 10^−6^ is used as a potential IV related to each exposure trait.^[16]^ SNPs related to the outcome variable (*P* < .05) are excluded. To avoid the influence of linkage disequilibrium (*R*^2^ < 0.001, window size = 10,000 kb), a clustering process is performed. The Mendelian randomization pleiotropy residual sum and outlier (MR-PRESSO) test is used to detect horizontal pleiotropy, and the pleiotropic effect is eliminated by removing outliers. In summary, according to the *P*-value of the MR-PRESSO test, SNPs are sorted in ascending order and the remaining SNPs are removed 1 by 1 until there is no pleiotropy (MR-PRESSO overall test *P*-value > .05).^[17]^ The strength of the selected SNPs is evaluated using the *F* statistic, where SNPs with an *F* statistic < 10 are excluded to avoid weak instrument bias in MR analysis. The statistical formula for *F* is *F* = [*R*^2^ × (n − *k* − 1)]/[*k* × (1 − *R*^2^)], where *R*^2^ is the portion of exposure variance explained by the IV, n is the sample size, and *k* represents the number of IVs.^[18]^ IVs with stronger associations with the outcome than the exposure are removed through Steiger filtering.

### 2.4. Two-sample Mendelian randomization

The MR method is employed to assess the causal relationship between artificial sweeteners and CHD. For exposures including multiple IVs, inverse variance weighting (IVW), maximum likelihood, MR-Egger, weighted median, and weighted mode methods are used to infer causality. IVW, as the preferred statistical method, provides the highest statistical power. IVW uses meta-analysis to combine the Wald ratio estimates of each IV in a meta-analysis, with the intercept constrained to 0.^[19]^ In the absence of horizontal pleiotropy, IVW can provide an unbiased causal estimate. When heterogeneity exists, a random-effects IVW test provides a more conservative and robust estimate; otherwise, a fixed-effects model is used. Similar to IVW, the maximum likelihood method assumes a linear relationship between exposure and outcome.^[20]^ MR-Egger validates the existence of multiple horizontal effects; when pleiotropy is present, it can provide an effective causal estimate. Even when up to 50% of the IVs are invalid, the weighted median can provide an effective causal estimate.^[21]^ Sensitivity analyses are conducted to assess the robustness of the causal relationship. To investigate horizontal pleiotropy, MR-Egger regression is utilized. Cochran’s *Q* test is employed to evaluate heterogeneity among IVs. Additionally, a leave-one-out sensitivity analysis is performed to determine whether any single SNP significantly affects the causal estimate. The MR Steiger analysis is applied to ascertain the potential direction of the causal relationship between the exposure and the outcome. All MR analyses are executed using R software (version 4.3.1; Vienna, Austria) with the ‘TwoSampleMR’ (version 0.5.7; https://github.com/MRCIEU/TwoSampleMR) and ‘MR-PRESSO’ (version 1.0; https://github.com/rondolab/MR-PRESSO) packages.

### 2.5. Reverse Mendelian randomization analysis

To examine whether CHD has a causal effect on the consumption of recognized artificial sweeteners (PIVW < 0.05), a reverse MR analysis was conducted. In this analysis, SNPs associated with CHD were utilized as IVs, with CHD serving as the exposure and artificial sweeteners as the outcome. The methodology used for the reverse MR analysis closely parallels that of conventional MR analysis.

### 2.6. Mediation analysis

Mediation analysis aims to assess the pathway from exposure to outcome through mediation, which helps explore the potential mechanisms by which exposure affects the outcome.^[22]^ Subsequently, multivariable Mendelian randomizationwas performed, in which insulin sensitivity and artificial sweetener intake were modeled simultaneously. This allowed estimation of the independent effect of insulin sensitivity on CHD – after adjusting for genetically predicted artificial sweetener exposure – yielding β(B). This approach helps determine whether the pathway linking sweetener intake to CHD operates, at least in part, through insulin sensitivity. The mediation effect is determined through a 2-step MR process: mediation effect = β(A) × β(B). The total influence of artificial sweeteners on CHD is derived from the earlier 2-sample MR, with the direct effect calculated as (total effect − mediation effect). The mediation proportion is calculated using the following formula: mediation proportion = (mediation effect/total effect) × 100%. The 95% confidence intervals (CIs) of the mediation effect and mediation proportion are estimated using the Delta method.^[22]^ The findings allow us to categorize the identified mediators into various levels of evidence. A triangular relationship indicates a causal link among the exposure, outcome, and mediator, suggesting that a causal relationship exists not only between exposure and outcome but also between mediator and outcome, as well as between exposure and mediator.

## 3. Results

### 3.1. Association of artificial sweeteners with CHD

**Figure 2.**
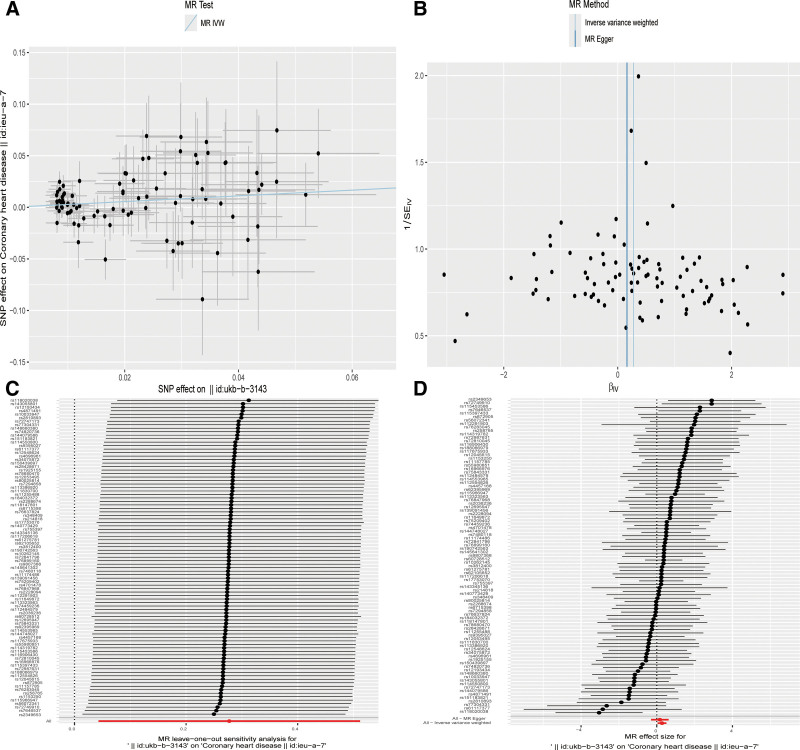
Scatter plots and “leave-one-out” results of genetic correlation between artificial sweeteners and CHD by different MR analysis methods. (A) Scatter plot. (B) Funnel plot. (C) Leave-one-out method. (D) Forest plot. CHD = coronary heart disease, IVW = inverse variance weighting, MR = Mendelian randomization, SNP = single nucleotide polymorphism.

**Figure 3.**
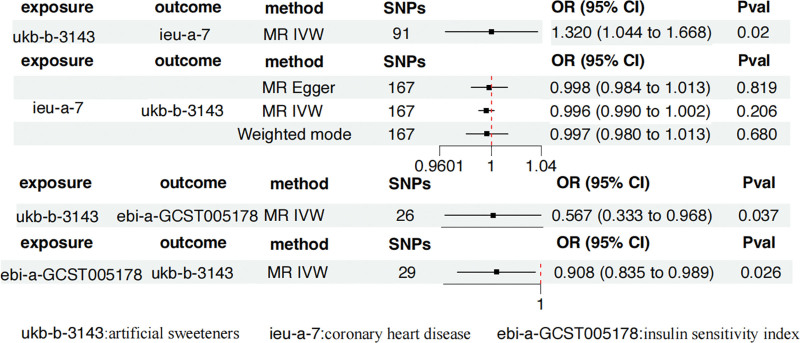
Two-sample MR analysis results. CI = confidence interval, IVW = inverse variance weighting, MR = Mendelian randomization, OR = odds ratio, SNP = single nucleotide polymorphism.

After removing palindromic and ambiguous SNPs, SNPs without proxies, and SNPs with incorrect causal directions identified by MR Steiger filtering, there are 103 SNPs in artificial sweeteners and 91 SNPs in CHD serving as IV. IVW, MR-Egger, simple mode, weighted mode, and weighted median regression are used to estimate the causal relationship between genetically predicted artificial sweeteners and CHD (Fig. 2). Among all MR methods, the IVW method supports a positive correlation between artificial sweeteners and CHD. The odds ratio (OR) per SD increase in IVW for CHD patients is 1.320 (95% CI, 1.044–1.668), *P* = .02. However, the results of our MR analysis in Figure 3 show that there is no reverse causal relationship between CHD and artificial sweeteners (that is, genetically predicted CHD has no causal relationship with artificial sweeteners; OR is 0.996 [95% CI, 0.990–1.002], *P* = .206).

### 3.2. Association of artificial sweeteners with insulin sensitivity index

**Figure 4.**
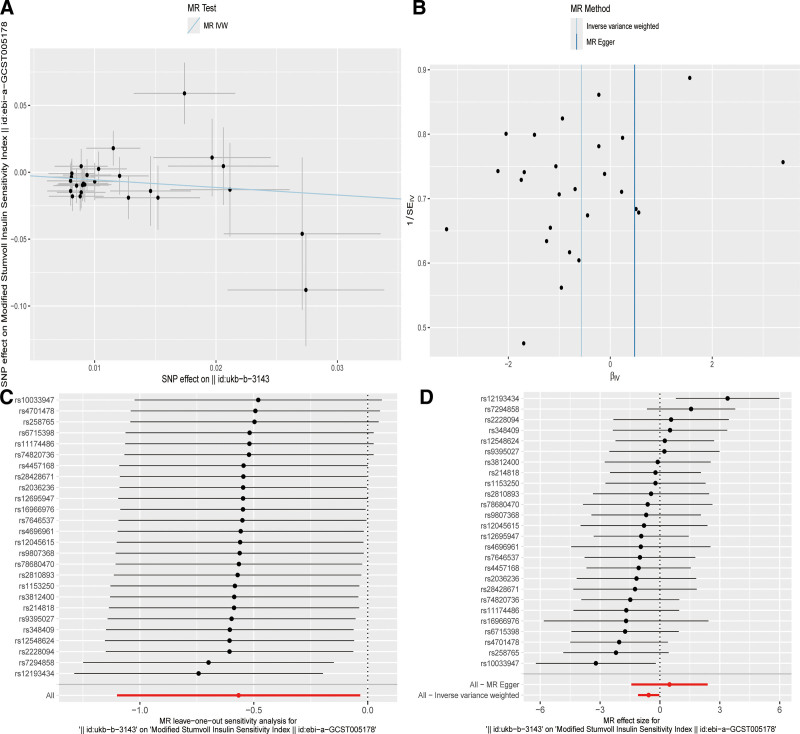
Scatter plots and “leave-one-out” results of genetic correlation between artificial sweeteners and insulin sensitivity index by different MR analysis methods. (A) Scatter plot. (B) Funnel plot. (C) Leave-one-out method. (D) Forest plot. IVW = inverse variance weighting, MR = Mendelian randomization, SNP = single nucleotide polymorphism.

After removing palindromic and ambiguous SNPs, SNPs without proxies, and SNPs with incorrect causal directions identified by MR Steiger filtering, we extracted a total of 26 genome-wide significant SNPs as IVs. According to the IVW method, it was found that genetically predicted artificial sweeteners are negatively correlated with the risk of insulin sensitivity index (OR by IVW method is 0.567; [95% CI, 0.333–0.968], *P* = .037). The results are shown in Figures 3 and 4.

### 3.3. Association of insulin sensitivity index with CHD

**Figure 5.**
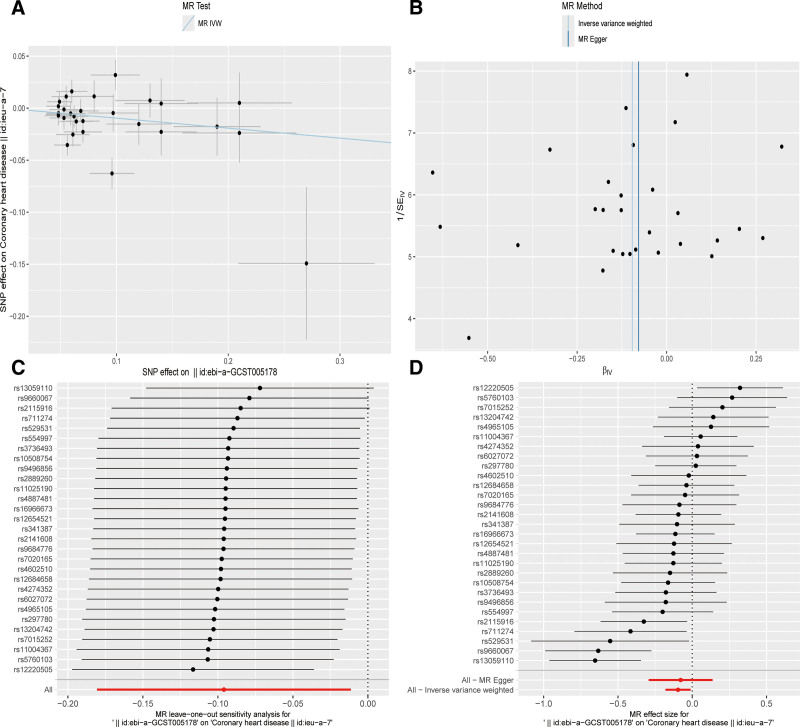
Scatter plots and “leave-one-out” results of genetic correlation between artificial insulin sensitivity index and CHD by different MR analysis methods. (A) Scatter plot. (B) Funnel plot. (C) Leave-one-out method. (D) Forest plot. IVW = inverse variance weighting, MR = Mendelian randomization, SNP = single nucleotide polymorphism.

As shown in Figure 3, with the IVW method, the genetically predicted insulin sensitivity index was significantly and negatively correlated with CHD (OR = 0.908, 95% CI, 0.835–0.989; *P* = .026). The results are shown in Figure 5.

### 3.4. Correlation between artificial sweeteners mediated by insulin sensitivity index and CHD

**Figure 6.**
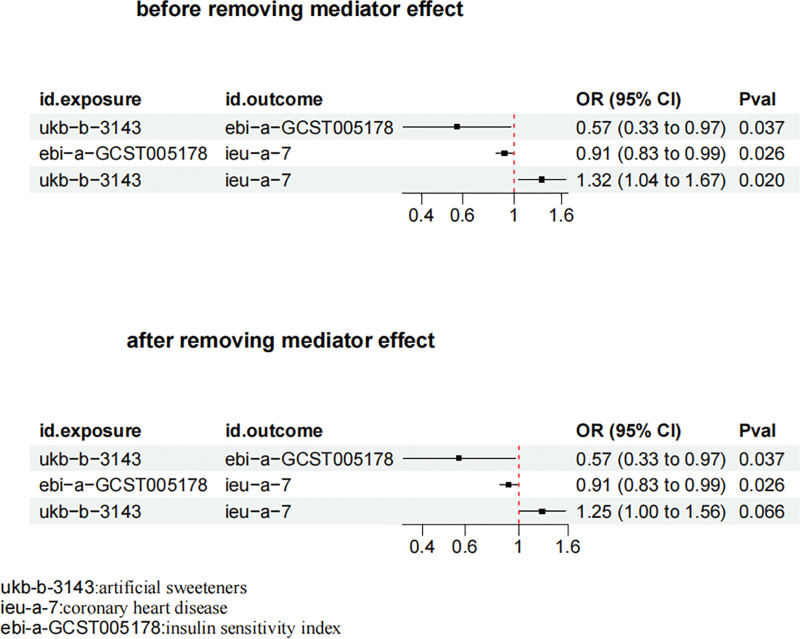
Correlation between artificial sweeteners mediated by insulin sensitivity index and CHD. CI = confidence interval, CHD = coronary heart disease, OR = odds ratio.

We analyzed insulin sensitivity index as a mediator of the correlation between artificial sweeteners and CHD. We found that the intake of artificial sweeteners is associated with an increase in insulin sensitivity index, and an increase in insulin sensitivity index is in turn associated with an increased risk of CHD. As shown in Figure 6, after removing insulin sensitivity index as a mediator, the intake of artificial sweeteners is no longer a risk factor for CHD (OR = 1.25, 95% CI, 1.00–1.56; *P* = .066; mediation ratio = 19.61%; Table S1, Supplemental Digital Content, https://links.lww.com/MD/R496).

### 3.5. Sensitivity analysis

Sensitivity analyses – including MR-Egger intercepts, Cochran’s *Q* tests, funnel plots, and leave-one-out analyses – revealed no evidence of substantial pleiotropy or heterogeneity. Causal estimates remained stable after removal of individual SNPs, confirming robustness (Table 1).

**Table 1:** Sensitivity analysis, including heterogeneity test and horizontal pleiotropy test.

Exposure	Outcome	Heterogeneity test (MR‒Egger)			Heterogeneity test (IVW)			Horizontal pleiotropy test (MR‒Egger)	
		Cochrane’s Q	Q_df	Q_pval	Cochrane’s Q	Q_df	Q_pval	Intercept	Pval
ieu-a-7	ukb-b-3143	205	165	0.02	205	166	0.022	0	0.732
ukb-b-3143	ieu-a-7	77.1	89	0.811	77.4	90	0.825	0.002	0.572
ukb-b-3143	EBI-A-GCST005178	22.3	24	0.563	23.5	25	0.547	-0.011	0.27
EBI-A-GCST005178	ieu-a-7	51	27	0.003	51.1	28	0.005	-0.001	0.861

Note: ukb-b-3143:artificial sweeteners, ieu-a-7:coronary heart disease, and ebi-a-GCST005178:insulin sensitivity index.

## 4. Discussion

This study provides compelling genetic evidence supporting a causal relationship between genetically predicted intake of artificial sweeteners and the risk of CHD. Using a 2-sample MR framework, we found that higher genetically determined artificial sweetener intake was associated with an increased risk of CHD, and that part of this effect was mediated through impaired insulin sensitivity. These findings address the long-standing uncertainty arising from observational studies, which have often been limited by residual confounding, reverse causation, and measurement inaccuracies in dietary self-report. By design, our MR approach minimizes these biases and strengthens the inference of causality.

Importantly, our study was specifically designed to evaluate whether artificial sweetener intake exerts a causal impact on CHD, rather than reflecting confounded or reverse associations. Consistent with this objective, the primary MR analysis demonstrated that each genetically predicted SD increase in artificial sweetener intake was associated with a significantly higher risk of CHD (OR 1.320, 95% CI, 1.044–1.668). The reverse MR analysis showed no evidence that genetic liability to CHD increased artificial sweetener intake, thereby supporting a unidirectional causal pathway from artificial sweetener exposure toward CHD development.

Artificial sweeteners, commonly utilized as substitutes for sugar, have generated ongoing debates concerning their impact on CHD. CHD is characterized by the narrowing of coronary arteries due to plaque buildup and remains a leading cause of mortality globally. The etiology of CHD is multifactorial, with dietary factors, particularly sugar consumption, being implicated in its development. Observational studies have suggested a positive association between the consumption of sugar-sweetened beverages and the risk of CHD.^[23,24]^ Artificial sweeteners are substances that provide sweetness with low or even no calories, serving as substitutes for traditional sugars.^[25]^ Notable examples of artificial sweeteners include aspartame, sucralose, acesulfame potassium, and steviol glycosides. Currently, the food industry often employs these sweeteners to meet consumer demand for low-sugar or sugar-free alternatives. For individuals with diabetes and those needing to manage their blood sugar levels, artificial sweeteners generally do not cause a rapid increase in blood glucose, thereby providing advantages for glucose control. However, studies have shown that long-term consumption of artificial sweeteners may affect the body’s blood sugar regulation mechanism, and the specific impact remains controversial.^[9,26]^ Additionally, several research papers have indicated that prolonged and excessive intake of artificial sweeteners could be linked to various health concerns, including cardiovascular diseases.

Our study demonstrated that genetically predicted higher intake of artificial sweeteners is associated with an increased risk of CHD. Rather than reiterating numerical estimates, this finding reinforces the central result observed in the MR analysis and highlights a potential adverse cardiovascular impact of long-term sweetener exposure. Based on previous studies, we suggest that insulin sensitivity index may serve as a key mediator between artificial sweeteners and CHD. We further identified insulin sensitivity as a potential mediator linking artificial sweetener intake to CHD risk. Genetically predicted higher sweetener exposure was associated with impaired insulin sensitivity, which in turn contributed to elevated CHD risk. After accounting for this pathway, the direct effect of sweeteners on CHD was substantially attenuated, supporting a partial mediation mechanism.

This research represents the first application of a comprehensive MR framework to examine the causal relationships involving artificial sweeteners, the insulin sensitivity index, and CHD. Moreover, by employing a 2-step MR approach and conducting mediation analysis, a pathway has been identified that connects artificial sweeteners to CHD via the insulin sensitivity index. To enhance the robustness of the MR findings, this study included a range of sensitivity analyses. However, certain limitations must be acknowledged. Firstly, the lack of demographic variables, such as age and gender, in the primary research restricts the potential for further subgroup analyses. Secondly, the majority of participants in the GWAS are of European descent, which limits the generalizability of the findings to other demographic groups. Additionally, while the MR approach is instrumental in evaluating causal relationships between exposure factors and outcomes, these findings require further validation through additional experimental and clinical research. Finally, it should be noted that the associations identified in this study reflect genetically mediated pathways rather than the direct physiological effects of actual dietary sweetener consumption. Therefore, caution is warranted when extrapolating these genetic estimates to real-world sweetener intake behaviors or dietary recommendations.

## 5. Conclusion

This study provides genetic evidence supporting a causal relationship between genetically predicted intake of artificial sweeteners and the risk of CHD. These findings reflect the effects of lifelong genetic predisposition to higher sweetener consumption rather than the direct physiological impact of actual dietary intake. Insulin sensitivity appears to partially mediate this relationship, offering insight into potential metabolic pathways linking sweetener exposure to cardiovascular risk. While our results strengthen the causal inference, they should be interpreted with caution when translating to real-world sweetener consumption patterns or dietary recommendations.

## Acknowledgments

We would like to thank all the authors who made outstanding contributions to this study.

## Author contributions

**Conceptualization:** Shu Sun, Yonggeng Zhang, Song Yi.

**Methodology:** Song Yi.

**Supervision:** Song Yi.

**Visualization:** Shu Sun, Song Yi.

**Writing – original draft:** Song Yi.

**Writing – review & editing:** Song Yi.

## Supplementary Material
