# Supplementary material for: Effects of artificial sweeteners on coronary heart disease: A 2-way 2-sample Mendelian randomization study and mediation analysis
Source: Medicine (Baltimore). 2026 Mar 13;105(11):e47656. doi: 10.1097/MD.0000000000047656 (PMC12991691; doi:10.1097/MD.0000000000047656)
Supplement: Supplementary file 1 [file medi-105-e47656-s001.docx]

**mediation effect**

| id.exposure | id.outcome | outcome | exposure | method | nsnp | b | se | pval | lo_ci | up_ci | or | or_lci95 | or_uci95 |
| --- | --- | --- | --- | --- | --- | --- | --- | --- | --- | --- | --- | --- | --- |
| ukb-b-3143 | ebi-a-GCST005178 | Modified Stumvoll Insulin Sensitivity Index \|\| id:ebi-a-GCST005178 | \|\| id:ukb-b-3143 | MR IVW | 26 | -0.56672546 | 0.272322979 | 0.037426734 | -1.100478499 | -0.032972422 | 0.567380309 | 0.332711843 | 0.967565243 |
| ebi-a-GCST005178 | ieu-a-7 | Coronary heart disease \|\| id:ieu-a-7 | \|\| id:ebi-a-GCST005178 | MR IVW | 29 | -0.095973235 | 0.043151746 | 0.026142593 | -0.180550657 | -0.011395814 | 0.908488331 | 0.834810391 | 0.988668872 |
| ukb-b-3143 | ieu-a-7 | Coronary heart disease \|\| id:ieu-a-7 | \|\| id:ukb-b-3143 | MR IVW | 91 | 0.277414007 | 0.119626562 | 0.020395032 | 0.042945946 | 0.511882069 | 1.319712628 | 1.043881467 | 1.668428338 |
| Mediation effect = β(A) × β(B). |  |  |  |  |  |  |  |  |  |  |  |  |  |
| Mediation ratio = (mediation effect/total effect) × 100% |  |  |  |  |  |  |  |  |  |  |  |  |  |
| β(A) =-0.566725460328662 |  |  |  |  |  |  |  |  |  |  |  |  |  |
| β(B)=-0.0959732352344179 |  |  |  |  |  |  |  |  |  |  |  |  |  |
| Mediation effect = β(A) × β(B)=0.0544 |  |  |  |  |  |  |  |  |  |  |  |  |  |
| Mediation ratio = (mediation effect/total effect) × 100%=19.61% |  |  |  |  |  |  |  |  |  |  |  |  |  |
| total effect=0.277414007148295 |  |  |  |  |  |  |  |  |  |  |  |  |  |
